# Are We Moving Too Fast?: Representation of Speed in Static Images

**DOI:** 10.5334/joc.404

**Published:** 2025-01-06

**Authors:** Irmak Hacımusaoğlu, Neil Cohn

**Affiliations:** 1 Department of Communication and Cognition, Tilburg School of Humanities and Digital Sciences, Tilburg University, The Netherlands

**Keywords:** visual language, visual lexicon, subjective speed, depicted motion, motion events

## Abstract

Despite pictures being static representations, they use various cues to suggest dynamic motion. To investigate the effectiveness of different motion cues in conveying speed in static images, we conducted 3 experiments. In Experiment 1, we compared subjective speed ratings given for motion lines trailing behind movers, suppletion lines replacing parts of the movers and backfixing lines set in the background against the baseline of having no extra cue. Experiment 2 was a replication of the first experiment with an addition of several motion lines considering the effect of quantity on conveyed speed. Experiment 3 then examined the actual time assessments of each cue and bare objects indicated for movers to complete their paths. Our results showed that motion cues vary in their effectiveness in depicting speed, with some influence from proficiency in reading manga. Motion lines, which index the path being traversed, remained less effective than suppletion and backfixing lines, which we argue encode the speed component of motion rather than directionality. However, adding more motion lines intensified the perceived speed of the movers. These static cues also influenced the actual time durations individuals indicated for fictitious motion events, in line with the subjective speed ratings. Altogether, our results suggest that different aspects of motion can be captured by different cues, and that the effectiveness of cues might be modulated by exposure to such patterns, in line with the premises of a visual lexicon view.

## General introduction

**Figure 1:**
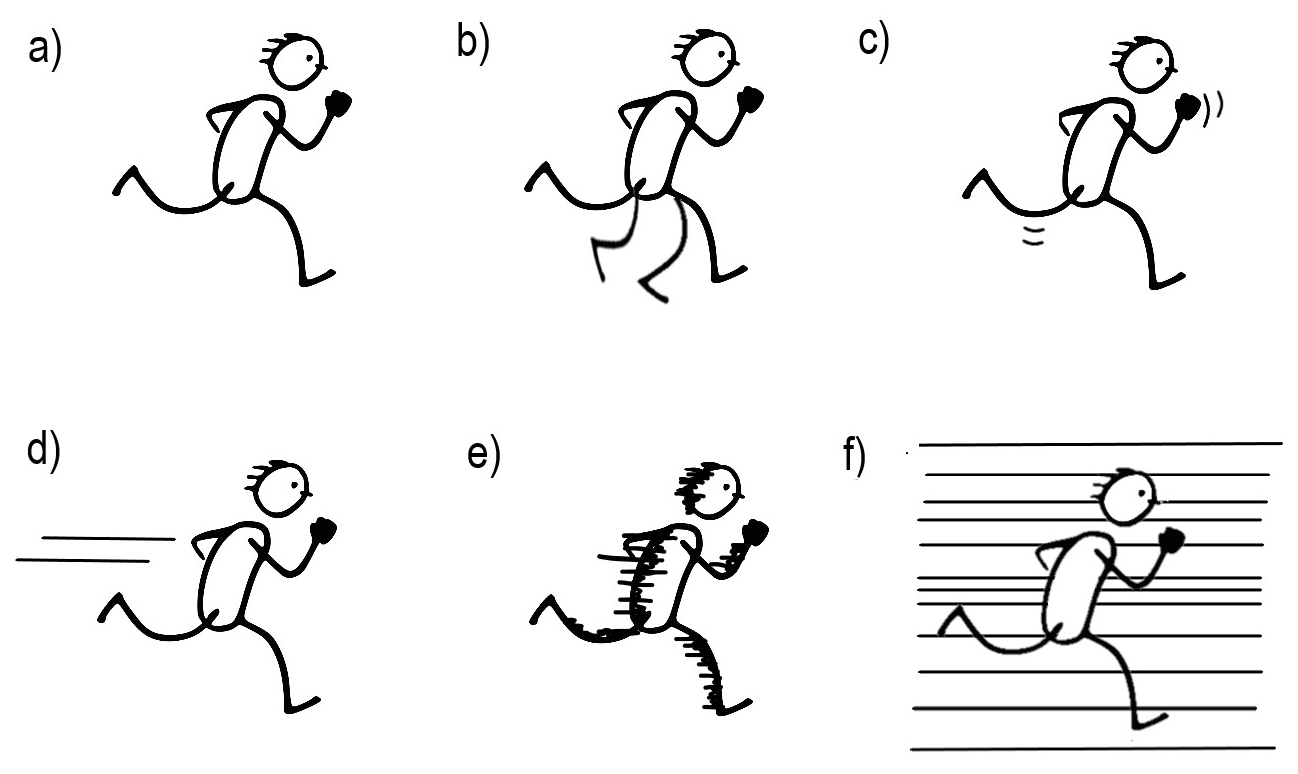
Lexical items that give a sense of movement in two-dimensional images **a)** postural cue **b)** repetition of (parts of) the mover **c)** contour lines mimicking the mover’s contours **d)** motion lines trailing behind the mover **e)** suppletion lines covering parts of the mover **f)** backfixing lines set in the background, behind the mover (Hacımusaoğlu & Cohn, 2023).

While pictures are inherently static, it is possible to convey dynamic information such as motion despite their two-dimensional nature. Depicting figures in poses or postures (Figure 1a) may convey an action that gives a sense of movement (Kourtzi & Kanwisher, 2000), while other methods include drawing lines trailing behind a mover, attaching several lines to the background, or replacing parts of the mover for lines (Hacımusaoğlu & Cohn, 2023). Among these cues, motion lines (also called action or speed lines)—the lines drawn behind the movers to indicate a traversed path—have received attention from various disciplines such as psychology, art, and linguistics (Burr, 2000; Carello et al., 1986; Cohn & Maher, 2015; Cutting, 2002; Hacımusaoğlu & Cohn, 2022; Kawabe & Miura, 2008; Kim & Francis, 1998; McCloud, 1993). Prior works have shown that motion lines enhance motion comprehension through clarifying the direction or path of motion (Francis & Kim, 1999; Kawabe & Miura, 2006) and influence the perceived speed (Carello et al., 1986; Gillan & Sapp, 2005; Hayashi et al., 2012). However, other motion cues in static images remain understudied, and such cues have not been compared against each other for their relative effectiveness in conveying motion or speed.

First, motion lines index a traversed path (as in Figure 1d), with the beginning of the lines marking the starting point of action and the end of the lines indicating the endpoint, relative to the mover’s current position. In this sense, motion lines can depict several moments at once. Previous research showed that, compared to no depicted motion lines, the presence of lines enhances comprehension of motion (e.g., Cohn & Maher, 2015; Ito et al., 2010), clarifies otherwise ambiguous directions of motion (Francis & Kim, 1999), and influences memory for the location of a given object in the direction suggested by motion lines (Kawabe et al., 2007). Besides path information, motion lines can also modify manner or characteristics of motion, such as speed (Hacımusaoğlu & Cohn, 2022). These intuitions even extend to children, who judged the depiction of runners as faster when presented with lines than without lines (Carello et al., 1986) and similar findings have been shown for interpretations of walking figures (Gross et al., 1991).

However, despite children’s interpretations, the understanding that those lines express motion does not come for free. Children progress across a developmental trajectory in understanding motion lines as a cue for movement (Brooks, 1977; Goodnow, 1978) beyond basic postural information (Friedman & Stevenson, 1975). Likewise, cross-cultural research has suggested that people without exposure to motion lines do not understand them as signaling motion (Duncan et al., 1973; Kennedy & Ross, 1975). Also, people who are experienced comic readers tolerate a lack of lines better than people who are less exposed to comics and thus motion lines (Cohn & Maher, 2015).

The advantages of lines for motion perception led to the idea that motion lines act analogous to motion streaks found in primary visual cortex that disambiguate the motion direction (Burr, 2000; Burr & Ross, 2002). Yet, comprehension of motion lines varies based on proficiency and cross-cultural exposure, going against the idea that motion lines are purely biologically based visual percepts. Other work has emphasized motion lines as learned metaphors (Forceville, 2011; Kennedy, 1982). However, neither perceptual nor metaphorical interpretations have considered further cues that are used to convey motion. Hence, despite being no consensus on how motion lines derive their meaning (see a review by Hacımusaoğlu & Cohn (2023)), they are likely to be stored in people’s visual lexicon together with other motion cues (Cohn, 2013) suggested by developmental and cross-cultural research.

A broader visual lexicon is suggested to involve several lexical items (Figure 1a-f) to convey motion (Cohn, 2013; McCloud, 1993). For instance, movement can be depicted through attaching several parallel lines to the background, behind a moving figure or object (as in Figure 1f). Like motion lines, these “backfixing lines” also gain meaning through being attached to a root or stem i.e., set in the background. While motion lines clarify the path traversed by indexing where the object once was, these lines only depict the mover in the middle of action, at a single moment. All a viewer would understand is that the mover is at the midpoint of the path’s trajectory, without any indication of a starting point or an endpoint. Thus, the direction of motion remains unclear (e.g., it might be from left to right, or vice versa). This is the case unless a postural cue indicates the direction being faced, as in Figure 1f, which does not rule out an interpretation that the figure is running backwards, against the postural direction.

If not direction, what do backfixing lines tell us? Backfixing lines, mostly seen in Japanese manga, were argued as giving a subjective sense of speed as if their viewers move at the same speed as the mover being depicted in static images (McCloud, 1993). This might be akin to seeing the background being blurred to look like parallel lines if viewed from inside of a fast-moving vehicle. Since the traveler moves at the same speed as the vehicle, the background would seem like parallel lines. Similar lines also appear in the background when a moving object is captured by a camera that follows the object’s trajectory or rotates horizontally from a fixed point (Bordwell et al., 2010). Thus, the form of backfixing lines arguably has a resemblance to its meaning, i.e., it is iconic (C. S. Peirce, 1902), but which is subtle and conventionalized as a lexical item in a visual vocabulary over time. Given that, the “reduced” iconicity of backfixing lines might also help them to convey speed information, in addition to backfixing lines indexing only a midpoint of a path.

Only a few studies have examined the psychology of backfixing lines. Ito et al. (2010) compared figures with backfixing lines, motion lines, and no lines in terms of their effectiveness in conveying motion. Backfixing lines did not enhance the impression of motion unless they converged into a vanishing point. This finding might be related to an inability of typical parallel/lateral backfixing lines to express the motion direction along with the sense of speed. By comparison, if they converge, the vanishing point shows where the mover comes from, and the expanded lines clarify the future direction even without any postural information. Therefore, parallel backfixing lines might have remained ineffective in depicting motion. However, this study did not test their effectiveness in denoting speed. Another study examined backfixing lines directly in relation to speed. Gross et al. (1991) asked both 7–9 years old children and adults to judge running and walking figures with motion lines, backfixing lines, and no lines. Across two experiments, children differentiated backfixing lines from no lines, but adults only did so when more realistic pictures were used in Experiment 2. This result was discussed as children attributing extra meaning to the lines in the background that adults did not do. While adults considered lines in the background as background elements e.g., a wall, children *assumed* they were similar to motion lines. Yet, some children actually reported that these are devices seen in cartoons, suggesting they might have greater exposure to backfixing lines, rather than assuming a resemblance that is not there. Adults interpreting these lines as background elements might then hint at their lack of exposure to backfixing lines used mostly in Japanese manga. Adults only relied on lines in the background of realistic scenes to depict speed, which might also hint at the iconicity of backfixing lines, that is reduced due to abstraction and conventionalization as discussed. Altogether, backfixing lines do not function to clarify the direction of motion but their function to denote speed remains less studied.

Besides attaching lines to the mover or to the background, another method to signal motion is to replace parts of the mover for lines (as in Figure 1e). These relatively short “suppletion lines” cover parts of the mover (Hacımusaoğlu & Cohn, 2023) and are also mostly associated with Japanese manga (McCloud, 1993). Like motion lines, suppletion lines are also attached to the mover, orthogonal to the motion direction, but unlike motion lines, these lines typically do not extend across a distance since they cover parts of the mover itself. As a result, the starting point of the action’s path information remains unclear. Instead, similar to backfixing lines, suppletion lines depict the mover in the middle of action at a single moment. Since suppletion lines do not mark the full path being traversed either, their function may remain as conveying speed rather than the path information.

Also, as with backfixing lines, suppletion lines are likely to have some degree of reduced iconicity. Suppletion lines are a graphic effect similar to motion blurs that result from capturing the motion of fast objects with a camera that cannot record the object at that speed (Navarro et al., 2011). In other words, blurring appears on photographs of moving objects because the camera itself is static but the thing being captured is in motion, and suppletion lines are akin to a line-based graphical depiction of this blurring. Like how direction is less overt in suppletion lines, directionality can remain ambiguous in blurs (Cutting, 2002). Given these, we might say speed is inherent to both suppletion lines and backfixing lines. However, despite their potential to convey motion in static images, compared to the many studies on motion lines, there is no empirical research on suppletion lines.

To sum up, several cues depict motion in static images, but most research has investigated only motion lines, with few studies comparing across motion cues. Therefore, we conducted three experiments to compare how all three motion cues and bare objects convey speed. In the first two experiments, participants assessed the speed of graphic objects based on the cues attached to them (Experiment 1) and the quantity of lines depicted (Experiment 2). In Experiment 3, we asked whether different cues would result in variation of participants’ actual response times for assessing the speed of graphic objects. Altogether, these studies aimed to assess whether different motion cues influence the conveyed speed and thus time duration differently. Overall, we predicted differences to arise between motion cue types, especially compared to depictions of movers with only objects and no additional cues.

## Experiment 1: Speed-time judgment of motion cues 1

Our first experiment aimed to assess the relative judgement of speed conveyed by different motion cues. Previous research demonstrated that motion lines trailing behind movers can modify their perceived speed. Even young children rated running figures as faster if presented with lines (Carello et al., 1986). Older children also produced lines to depict fast movement (Goodnow, 1978) and both children and adults judged moving figures with the presence of lines as faster compared to without lines, regardless of the stimuli type (Gross et al., 1991).

Backfixing lines however led to mixed results. When more cartoony stimuli were used, children chose motion lines over no lines and judged both motion lines and backfixing lines as fast. Adults on the other hand rated motion lines as showing faster action than backfixing lines. In photograph-like stimuli, again both 7- and 9-year-olds judged the presence of lines (either type) as faster than lack of lines, and it was the case regardless of the movement type. Also, both age groups viewed running figures with motion lines as faster than with backfixing lines, but for walking figures only older children did so. Adults again preferred motion lines over backfixing lines for photographic stimuli, but only for figures in running posture while the presence of lines did not matter for walking figures (Gross et al., 1991).

Given these limited and mixed findings, Experiment 1 compared motion lines (two parallel lines) and backfixing lines in terms of their ability to denote speed. To eliminate the additive effect of the posture (e.g., walking vs. running) on perceived speed, we used objects instead of animate figures. In addition to motion lines and backfixing lines, we also added suppletion lines that replace parts of the movers to depict motion.

We expected objects shown on its own with no extra motion cue to be the slowest. This is because objects would be in a frozen state without any cue on them, given that they do not have postural information inherent to indicate motion direction or speed. Then, because motion lines index the traversal of a path and clarify motion direction rather than showing the object in the middle of presumably fast action, we expected them to be less effective in conveying speed than backfixing and suppletion lines. In other words, given that backfixing and suppletion lines show the object in the middle of action without marking a full path, we predicted them to encode the speed component of motion better than motion lines. Finally, while no prior research compared backfixing and suppletion lines, objects with suppletion lines might be perceived faster than ones with backfixing lines, because the former depicts the motion from an objective viewpoint, i.e., viewers remain constant with suppletion lines, as opposed to backfixing lines that create the sense of a viewer moving at the same speed as the object. This aspect of suppletion lines could potentially heighten the assessed speed.

### Methods

#### Design and Stimuli

We created 16 single panels showing a mover and its goal using free images from cocomaterial.com. Stimuli consisted of different types of balls (e.g., football), objects (e.g., glass), land-based motor vehicles (e.g., cars) and food (e.g., fruits). Each type of mover constituted 25% of the stimuli. Based on the type of the mover, appropriate goals were selected such as a goalkeeper for balls and a monkey with open hands for fruits. Note that the presence of the goal itself might suggest movement by default, but this was intentional because otherwise it would not be an action scene and there would not be any so-called movers.

**Figure 2:**
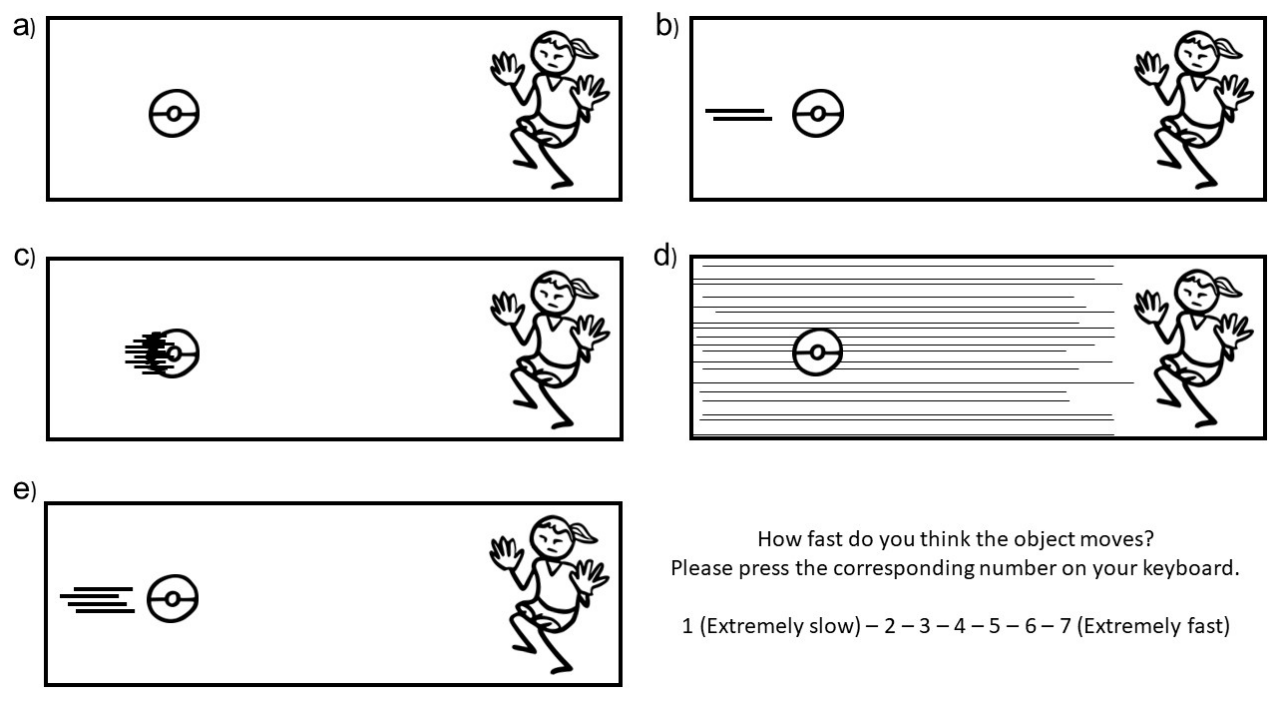
Motion cues of **a)** object-only condition where no additional cue appeared to indicate motion on the object itself **b)** motion lines as two parallel straight lines trailing behind the mover **c)** suppletion lines **d)** backfixing lines (in Experiments 1 and 2) and **e)** several motion lines (Experiment 2). Each panel comes with the rating question in the original trials. Stimuli were adapted from CocoMaterial images.

**Figure 3:**
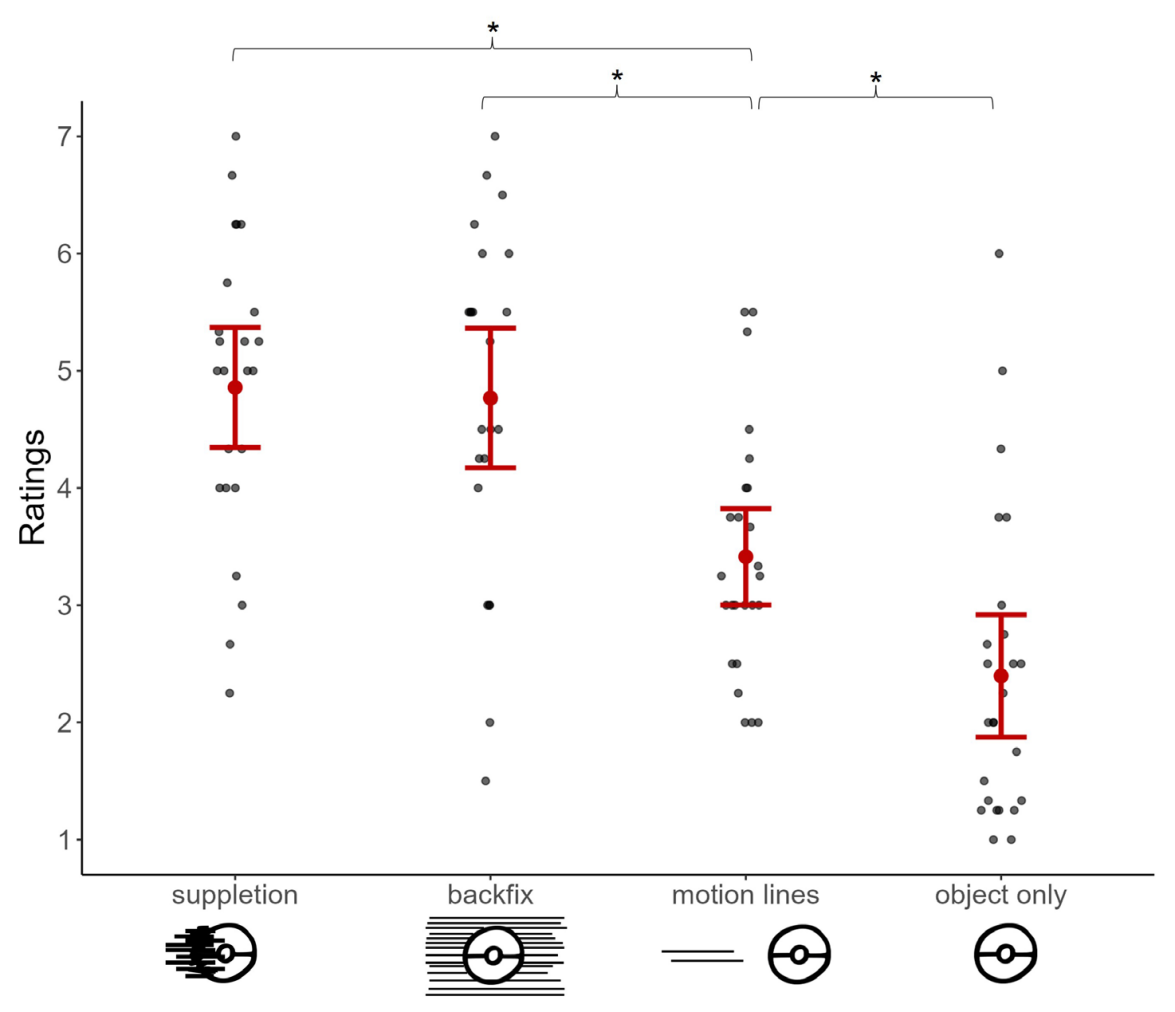
Subjective speed ratings (1 = extremely slow, 7 = extremely fast) averaged across each motion cue type. Each gray dot represents averaged ratings per participant while the red dot indicates the overall mean, and error bars show the standard errors. Asterisks (*) highlight the significant differences that arose between the tested contrasts.

To test the effect of motion cues on subjective speed judgment, we varied the base stimuli showing objects alone (object-only condition, Figure 2a) in three ways, by 1) adding two straight parallel lines trailing behind the mover (motion lines, Figure 2b), 2) replacing parts of the objects for lines (suppletion lines, Figure 2c), 3), or attaching parallel lines in the background behind the mover (backfixing lines, Figure 2d). Constant distances were maintained both between the left border of the panel and the edge of the mover and the distance between the mover and motion lines. Also, the same number of lines were used with suppletion lines, but depending on the contour shape of the object, lines were accordingly attached in different places.

Altogether, our design resulted in 4 motion cue types. We manipulated all panels (16) for all conditions (4), resulting in 64 stimuli in total. With a Latin Square Design, we prepared 4 lists to prevent participants to see the same scenario in different conditions (e.g., if an apple appeared with motion lines, it did not appear with suppletion lines). This resulted in 16 stimuli per list. For each panel, the task was to indicate how fast the object moves using a Likert type scale from 1 (extremely slow) to 7 (extremely fast). We programmed all experiments in PsychoPy version 2022.2.4 (J. Peirce et al., 2019) and randomized trials per participant.

#### Participants

We recruited 25 participants^1^ during the 2022 Dutch ComicCon on a voluntary basis (female: 13, male: 11, other: 1, mean age: 25.28, range: 18–47). The experiment was approved by Tilburg University’s Research Ethics and Data Management Committee (on 11 November 2022 with the approval code REDC2022.63). All participants gave their informed written consent prior to the experiment stating that they can withdraw from the experiment any time without consequences.

To assess participants’ comic reading expertise, we used the Visual Language Fluency Index (Coderre & Cohn, 2023; Cohn, 2020). An average score for this metric would be 12, where scores below 7 are considered low, and those exceeding 20 are deemed high. In this experiment, VLFI scores of our participants indicated an average-level fluency, with a mean score of 14.43. Participants were also asked to indicate the languages they speak and their fluency in those languages.

#### Procedure

Participants took the experiment at a booth at the Dutch ComicCon by sitting in front of a laptop (screen display size 14 inches). After signing the informed consent and filling in the demographics forms (VLFI and language ability), participants began the experiment. The first screen welcomed them and provided instructions for the experimental task. Instructions were also explained verbally. To start the experiment, participants were instructed to click on the space bar and that the task was to rate how fast they think the object is moving toward its goal by clicking on the corresponding number on the keyboard. Once participants gave their rating on the subjective speed for the subsequent panel, they proceeded to the next panel automatically. After the completion of all trials, they received a thank you message on the screen with a brief explanation about the manipulations. We also debriefed participants verbally.

#### Data Analysis

We measured the speed judgment ratings (1 = extremely slow, 7 = extremely fast) and response times (measured in milliseconds) for each panel. Both ratings and response times were averaged across conditions per each participant. To remove the outliers, we used the method of Median Absolute Deviation or MAD (Howell, 2005) in which the average distance of each data point from the median was computed. We subtracted median + 2.5 MAD from raw response times and excluded response times exceeding this threshold. The data for all experiments can be found at its DataverseNL repository, https://doi.org/10.34894/S8LC85 (see the Data Accessibility statement for further details).

To examine the effect of cues on average speed ratings and response times respectively, we conducted two Linear Mixed-Effects Models and we set motion cue type (object only, motion lines, backfixing lines, suppletion lines) as the fixed factor and items and participant id as random factors. In case of a significant main effect, our planned contrasts compared motion lines to object only, backfixing and suppletion lines to motion lines, and suppletion lines to backfixing lines. Effect sizes (Cohen’s d) for each contrast was calculated dividing the contrast estimates by residual’s standard deviation. Finally, we looked at the relationship between overall VLFI scores and/or fluency in reading manga and each dependent variable through correlation analyses in an exploratory fashion. All analyses in all experiments were conducted using Jeffreys’s Amazing Statistics Program or JASP (JASP, 2024).

### Results

#### Ratings

The first analysis examined average subjective speed ratings, and showed a main effect of motion cue type, *F*(3, 293.94) = 77.057, *p* < .001. Planned contrasts revealed that suppletion lines and backfixing lines were both rated as conveying faster motion than motion lines (all *z*s > 7.6, *p*s < .001, *d*s = 1.18) as in Figure 3, but they did not differ from each other (*p* = 0.970, *d* = –0.01). Also, motion lines led to higher speed ratings than object-only condition (Figure 3), *z* = 5.1, *p* <.001, *d* = 0.79.

Our exploratory correlation analysis found no relationship between participants’ fluency in reading comics and their subjective speed ratings (*r* = 0.085, *p* = 0.406).

#### Response Times

We then examined the response times of motion cues, but found neither a main effect of cue type (*F*(3, 304.28) = 0.768, *p* = 0.513) nor a relationship to participants’ fluency scores (*r* = 0. 075, *p* = 0.463).

### Discussion

This experiment investigated the subjective perception of speed conveyed by different graphic motion cues. Overall, movers presented without any cue on them were judged as the slowest (see Figure 3) while both suppletion lines and backfixing lines were rated as conveying faster speed than motion lines. These results confirm our expectation that motion cues differ in their effectiveness in conveying speed. Moreover, no difference arose between response times for different cues, suggesting that the difficulty of making these judgements was similar across cues and it was the case regardless of participants’ fluency in reading comics.

The lower speed judgments given to objects with no extra cue substantiates that, objects lacking a postural cue would only seem in a frozen state and thus be perceived as the slowest. Also, motion lines were judged to convey faster speed than only objects presented alone. This finding supports the idea that motion lines clarify the direction of motion, but do not necessarily denote faster speed. By comparison, backfixing and suppletion lines that depict movers in the middle of action *without* marking the traversed path were rated as showing the fastest speed. This is also in line with our reasoning that these cues are likely to have reduced iconicity to convey faster speed, since they do not necessarily disambiguate the motion direction or mark the paths being traversed.

Overall, Experiment 1 confirmed the effect of motion cues on perceived speed as found in prior works (Carello et al., 1986; Gross et al., 1991) as well as the differences between motion cues in their functions to depict speed component of motion. However, one difference between these cues is that motion lines in this experiment used fewer lines than suppletion and backfixing lines. Indeed, the number of lines has been shown previously to modulate the perceived speed (Gillan & Sapp, 2005; Hayashi et al., 2012). Also, motion lines can vary from the two parallel straight lines used here, such as their shape or the number of lines being used. Relatedly, the mixed results found in the comparison of motion lines and backfixing lines would be further clarified with the addition of more lines. In particular, multiple motion lines have in some cases been found to be interpreted as faster than backfixing lines (Gross et al. 1991). Since more lines might add up on bare effect of having only two lines, Experiment 2 explored how an increase in the number of motion lines would reflect in their effectiveness in expressing speed relative to other motion cues.

## Experiment 2: Speed-time judgment of motion cues 2

Our second experiment aimed to compare the perceived speed of different motion cues and motion lines with varying amounts of lines. In prior work, higher speed ratings were given as the number of motion lines increased, but other cues like thickness of lines had no effect (Gillan & Sapp, 2005). In addition, Hayashi et al. (2012) used a prediction-motion paradigm in which they asked participants to estimate the speed of a ball that appeared on the screen for a standard duration with different number of motion lines attached, before disappearing and reappearing when participants pressed a button. The number of lines affected the speed estimations. However, because 5 motion lines resulted in quicker reaction time—and thus faster assessed speed—than a single line, but did not differ from 8 lines, their results did not indicate whether a linear relationship held between the number of lines and higher speed judgments, or if it merely involved exceeding a certain threshold of lines. Indeed, when children were asked to represent speed by drawing a slow- versus fast-moving person, not many children used motion lines but when they did, they depicted the faster person with a greater number of motion lines (Gross et al., 1991).

Given that motion lines mark the path being traversed, the presence of only two lines would be enough to clarify the direction. The addition of more lines might then add up on the speed component that is not directly captured by typical (only two) motion lines. Intensified meaning by the addition of more lines would be also in line with the linguistic notion that “more in form = more in meaning” (Lakoff & Johnson, 1980). Relatedly, similar to spoken languages, repetition can emphasize meaning in the visual-graphic modality (Hacımusaoğlu & Cohn, 2023) such as multiple heart shapes between a couple intensifying the strength of their love compared to having only one heart shape.

Experiment 2 therefore sought to confirm that more motion lines would lead to greater perceived speed, particularly relative to backfixing and suppletion lines. We again compared all stimuli with motion cues to one another and motion lines to objects presented alone. We predicted several motion lines to result in higher speed ratings than fewer motion lines. We had no prediction for whether the increase in the number of motion lines would minimize the differences found between motion lines and other cues.

### Methods

#### Design and Stimuli

The design was almost identical to Experiment 1, but we added one more motion cue (i.e., several motion lines) to assess a possible effect of quantity of motion lines on speed ratings. Here, instead of two parallel straight lines, four lines trailed behind the movers (see Figure 2e). Since the number of motion cue types increased to 5 in this experiment, to maintain equal numbers of stimuli per list, we increased the number of each mover to 5 as well (i.e., adding one more ball, object, vehicle, and food supplement). In total, 80 stimuli were created resulting in 16 stimuli per list.

#### Participants

Experiment 2 was conducted in a lab at Tilburg University with an ethics approval from the REDC (obtained on 20 March 2023 with the approval code REDC 2023.11). 30 participants were recruited through the participant pool, in exchange for course credit (female: 15, male: 15, other: 0, mean age: 21.4, range: 18–30). Their VLFI scores showed an average-level fluency in comic reading, with a mean score of 13.46. Participants were again asked to indicate the languages they speak and their fluency in those languages.

#### Procedure

Other than the change to a lab setting, the experimental procedure was identical to Experiment 1.

#### Data Analysis

After outlier removal (MAD), Linear Mixed-Effects Models were conducted on ratings and response times. Again, we set the motion cue type as the fixed factor but this time with 5 levels (object only, motion lines, several motion lines, backfixing lines, suppletion lines). Items and participants were used as random factors to eliminate the possible effect of strips and individual differences on dependent variables. In case of a significant main effect, our planned contrasts targeted each cue against one another. Objects alone were compared to motion lines. Exploratory correlation analyses were then performed on overall VLFI scores and/or fluency in reading manga and each dependent variable.

### Results

#### Ratings

**Figure 4:**
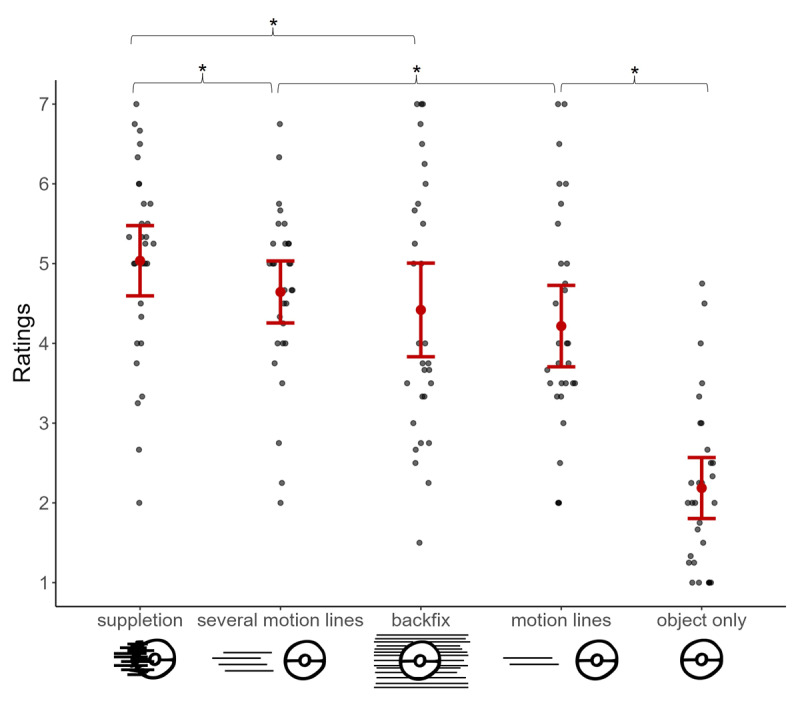
Subjective speed ratings (1 = extremely slow, 7 = extremely fast) given in Experiment 2 averaged across each motion cue type. Each gray dot represents averaged ratings per participant while the red dot indicates the overall mean, and error bars show the standard errors. Asterisks (*) highlight the significant differences that arose between the tested contrasts.

In the analysis of subjective speed ratings, we found a main effect of motion cue type, *F*(4, 477.38) = 78.333, *p* < .001. As shown in Figure 4, planned contrasts revealed that suppletion lines were rated as showing faster motion than both backfixing lines (*z* = 3.4, *p* = .003, *d* = 0.47) and several motion lines (*z* = 2.7, *p* = .019, *d* = 0.38). In turn, several motion lines conveyed faster speed than motion lines (*z* = 2.8, *p* = .019, *d* = 0.39) while motion lines were more effective in conveying speed than objects alone (*z* = 10.7, *p* < .001, *d* = 1.48). However, backfixing lines did not differ from several motion lines (*p* = 0.498, *d* = 0.09) and their difference from motion lines did not reach to the significance level (*p* = .064. *d* = 0.30).

Exploratory correlation analysis revealed no relationship between the ratings and VLFI, *r* = –0.025, *p* = 0.760.

#### Response Times

A main effect of motion cue type showed response times differed across participants based on the cues, *F*(4, 492) = 3.4, *p* = .009. Our planned contrasts indicated participants spent longer time to rate objects with backfixing lines than with several motion lines (*z* = 2.9, *p* = .026, *d* = 0.40) and motion lines (*z* = 3.05, *p* = .018, *d* = 0.13). None of other cues varied from each other, all *p*s > 0.171.

We found no correlation between participants’ general fluency scores and their response times (*r* = 0.083, *p* = 0.315) while further exploratory analysis on fluency specific to manga readership yielded a positive correlation (*r* = 0.216, *p* = .008). The more people read manga while growing up and currently, the longer it took for them to rate the given motion event scenarios in Experiment 2. Specifically, more fluent manga readers showed a trend toward having longer response times to rate motion lines (*r* = 0.355, *p* = .055).

### Discussion

This experiment looked at the perception of speed conveyed by different motion cues by further exploring several motion lines attached behind the objects. Like in Experiment 1, motion cues differed in their effectiveness in depicting speed while objects presented alone were perceived as the slowest (Figure 4). In addition to Experiment 1, the presence of more motion lines led to greater speed ratings than just having two lines trailing behind movers, in line with the expectations. Yet, several motion lines were not as speedy as suppletion lines but were comparable to backfixing lines. Finally, participants took longer to rate backfixing lines compared to both types of motion lines, but participants with greater exposure to Japanese manga tended to take a longer time to rate motion lines but not backfixing lines.

Furthermore, the addition of lines behind the movers was consistent with the prior work showing faster speed judgments as the number of lines increased. Given that two parallel lines would be enough to index the path being traversed already, extra lines might have highlighted the speed component of motion. This would align with how repetition can also be used to intensify meaning in other modalities (Hacımusaoğlu & Cohn, 2023). Also, increase in the number of motion lines made them comparable to backfixing lines, unlike just having two motion lines in Experiment 1. Yet, several motion lines were still judged to be slower than suppletion lines. This confirms the effectiveness of suppletion lines in conveying speed despite being less often used in comics than motion lines (Hacımusaoğlu & Cohn, 2022).

However, backfixing lines in this experiment also led to inconsistent results. While they were as fast as suppletion lines in Experiment 1, here they were rated as slower than suppletion lines. More unexpectedly, their difference from (only two) motion lines did not reach significance. This could be due to proficiency-related differences observed for processing of backfixing lines in Experiment 2 which was not present in Experiment 1. As corpus studies have shown that backfixing lines are more prevalent in manga (Cohn, 2023) and they convey speed from a subjective viewpoint by drawing the viewer into the scene, exposure or lack of exposure to such cues might influence how fast they are perceived depending on the sample. This may not have been fully captured by the individual correlations we conducted here.

**Figure 5:**
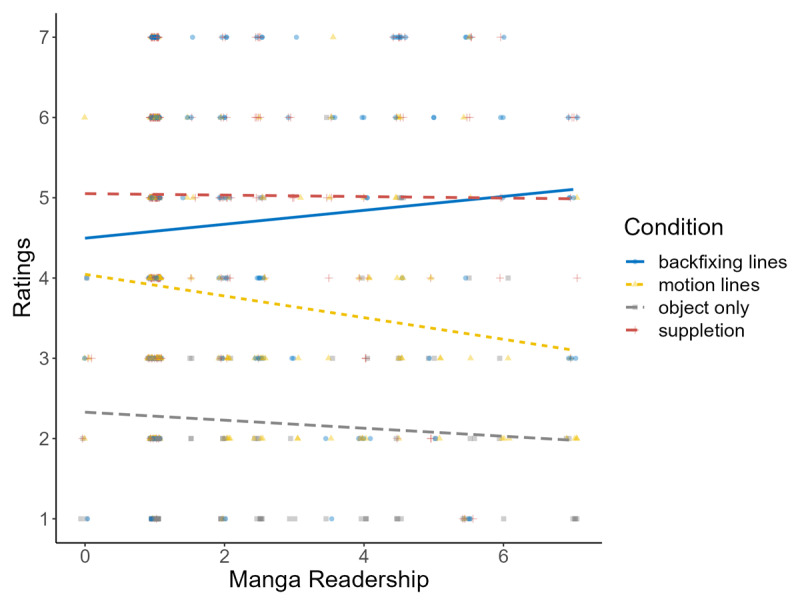
Y-axis shows the subjective speed ratings (1 = extremely slow, 7 = extremely fast) and x-axis shows participants’ fluency scores in reading manga (higher numbers indicate greater fluency levels) when the data from Experiments 1 and 2 were combined. Each line corresponds to a motion cue type that were present in both experiments (i.e., backfixing lines, motion lines, object only, suppletion lines). This graph shows the magnitude of the difference found between backfixing lines (blue line) and motion lines (yellow line) changes depending on participants’ experience levels in reading manga.

To resolve this discrepancy, we merged data from Experiments 1 and 2, excluding several motion lines, and added manga readership as a fixed factor and experiment id, items, participants as random factors in the model. Here, suppletion lines were perceived as faster than backfixing lines (*p* = .010, *d* = 0.27), which, in turn, were faster than motion lines (*p* < .001, *d* = 0.68). Additionally, manga readership yielded an interaction where, as shown in Figure 5, individuals with greater exposure to manga rated backfixing lines as faster compared to those with lower fluency in manga readership. The opposite pattern was observed for motion lines. This additional finding suggests that fluency in manga can modulate the perceived speed of backfixing lines and motion lines, while the effectiveness of suppletion lines remained consistently the highest.

## Experiment 3: Speed-time judgment of motion cues 3

Experiments 1 and 2 indicated that participants view motion cues as conveying different speeds of movement. Given this, we next asked: Does the fictive speed of motion cues affect the real-life timing of judging their speed? We adapted the design by Hayashi et al. (2012) who measured participants response times as an indication of speed estimation (see Experiment 2 introduction), who showed that estimation of cue speed was influenced by both the number and length of motion lines. They found movers with only one motion line led to slower response times and thus slower speed estimations than having more lines. Difference in speed estimations also emerged based on the length of line, as shorter motion lines resulted in longer reaction times thus slower speed than long ones.

Based on this, we presented objects in the starting point and then in the midpoint of a path and asked participants to indicate when they think the objects would complete their paths or arrive to their targets. We again compared different motion cues (motion lines, several motion lines, backfixing lines and suppletion lines) relative to having objects presented alone. Given that time and speed are inversely proportional to each other, we based our predictions on our findings of speed judgments in Experiments 1 and 2. Specifically, we expected suppletion lines to be perceived the fastest and thus they would involve the shortest time estimations. Also, because we found more motion lines to show faster movement in Experiment 2, here we expected several motion lines to yield shorter time estimations than motion lines. In turn, motion lines might take shorter than having objects only since the latter was by far the slowest in other experiments. Finally, because backfixing lines were judged as fast as suppletion lines in Experiment 1 but not in Experiment 2, they might place in between suppletion lines and several motion lines.

### Methods

#### Design and Stimuli

**Figure 6:**
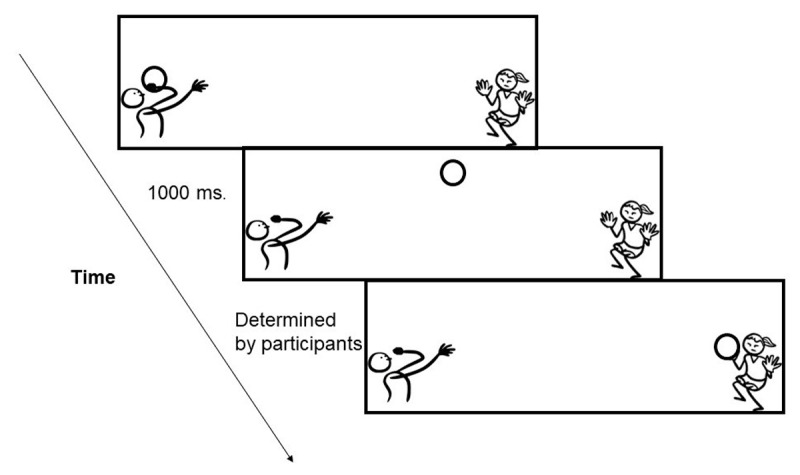
The design of Experiment 3. Between the first two frames showing the starting point (panel 1) and midpoint (panel 2) of the action, SOA was always constant as 1000 ms. Participants then pressed a button to indicate when the object would reach to its endpoint (panel 3), and we measured the real time estimates given for each cue between panels 2 and 3. The figure illustrates object-only condition.

We created 20 three-panel strips showing a person throwing a ball to another person, again using images from cocomaterial.com. As in Figure 6, the first panel depicted a person holding and about to throw a ball (the starting point or source), the second panel showed the ball in the middle of the air, and once the participants pressed a button, the final panel showed the moment that the ball reaching its goal i.e., another person catching it. This time the aim was to measure participants’ subjective judgment of *how long* it would take the mover to reach to its goal. In that sense, we adapted the design of Hayashi et al.., (2012) to retain the source/goal images on screen instead of making them disappear until participants responded. Since in real settings, visuals (e.g., comic panels) do not disappear, our adapted design aimed to be closer to the naturalistic way that visual communication operates. Also, to eliminate possible confounds of the possible relative speeds of different objects on subjective time estimation (e.g., cars move faster than apples), we only used different depictions of balls as stimuli in this experiment.

To test the effect of motion cues on participants’ time estimation, we again used the same cues as in Experiment 2 (motion lines, several motion lines, suppletion lines, backfixing lines) in addition to the object-only condition (no extra cue). Also, since the event of throwing a ball creates a curving path (instead of straight), curvy lines were used instead of straight lines in motion lines conditions (only two lines and several). The other depictions of motion types were similar to those in Experiments 1 and 2 given that backfixing and suppletion lines appear straight in actual comics regardless of the nature of the action. We manipulated all strips (20) for all conditions (5), and thus created 100 stimuli in total to result in 20 stimuli per list. The task was to determine when the ball would reach to its goal (see the procedure below).

#### Participants

We again recruited participants from the participant pool, with the requirement that they did not take part in Experiment 2. The study was approved by the REDC (same approval as Experiment 2), and 30 participants participated in Experiment 3 for course credit (female: 21, male: 9, other: 0, mean age: 20.63, range: 18–25). Their fluency in comic reading was around average, with a mean of 11.58. The procedure of collecting language data was identical to prior experiments.

#### Procedure

Experiment 3 also took place in the same lab setting as Experiment 2. Participants provided their written informed consent prior to the experiment and received the instructions on the screen. The task was slightly different than the previous two experiments. Participants received 3-panel strips instead of a single panel, with each panel being viewed one at time in the middle of the screen. At the first panel they viewed a character on the left side of the panel with a ball. The second panel then showed the ball in the middle of the air. The time passage between the first and second panel (Stimulus Onset Asynchrony or SOA) were kept constant as 1000 milliseconds for all stimuli. Next, participants were instructed to press the spacebar when they thought enough time passed for the ball to reach to the goalkeeper. They clicked on the spacebar when they thought the ball would reach to its goal and response times were measured. Afterwards, we debriefed participants both verbally and by providing the explanations on the screen.

#### Data Analysis

In this experiment, response time was measured between Panels 2 and 3, which indicated the participants’ subjective time estimation. Outliers were removed by using the method of MAD. To test the effect of motion cue type (object only, motion lines, several motion lines, backfixing lines, suppletion lines) on the estimated time durations, we conducted a Linear Mixed-Effects Model with motion cue type set as fixed factor and items and participant id as random effects factors. In case of a significant main effect, our planned contrasts compared each cue against one another. Objects alone were compared to motion lines. Again, additional correlation analysis was performed on VLFI scores and/or fluency in reading manga and the estimated time durations.

### Results

**Figure 7:**
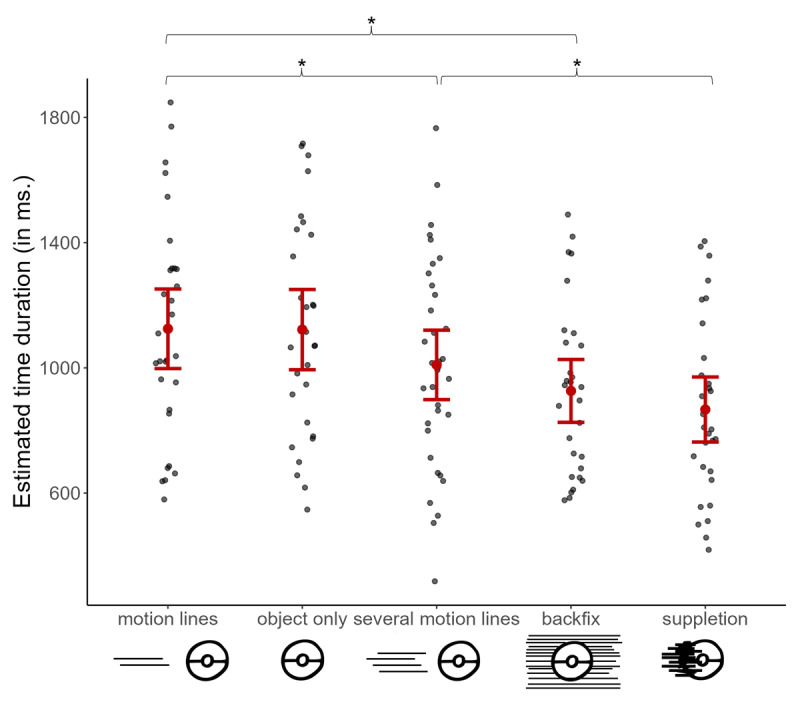
Estimated time durations averaged across motion cue types. Each gray dot represents each participant’s averaged time estimates per condition, while the red one showing the mean, and error bars show standard errors. Asterisks (*) highlight the significant differences that arose between the tested contrasts.

A main effect of motion cue type showed that subjective time estimation differed based on cues, *F*(4, 488.88) = 12.913, *p* < .001. Planned contrasts then demonstrated that both suppletion (*z* = –5.65, *p* < .001, *d* = –0.78) and backfixing lines (*z* = –4.23, *p* < .001, *d* = –0.59) led to shorter time estimation than motion lines but did not differ from each other (*p* = 0.336, *d* = –0.19), as shown in Figure 7. Suppletion lines also resulted in shorter time estimation compared to several motion lines (*z* = –2.98, *p* = .014, *d* = –0.41) while several motion lines took less time than motion lines (*z* = –2.6, *p* = .034, *d* = –0.37). Finally, several motion lines did not differ from backfixing lines (*p* = 0.336, *d* = 0.22), and motion lines and objects presented alone were comparable to each other (*p* = 0.866, *d* = –0.02).

A negative correlation between estimated time duration and fluency in reading comics (r = –0.207, *p* = .010) indicated that more fluent people estimated shorter subjective time passage for the given motion events.

### Discussion

Our final experiment examined the subjective estimation of the duration of movement based on different motion cues. We found cues differed in how they influenced the estimated time durations: First, suppletion lines were perceived as faster than motion lines regardless of their quantity, indicated by shorter time durations, while backfixing lines were found to be faster than motion lines but comparable to suppletion lines. Greater number of motion lines again led to faster speed than only two lines and was comparable to backfixing lines. In addition, a negative relationship appeared between time estimations and participants’ fluency in reading comics suggesting that more experienced comic readers indicated shorter time passage for the given motion events, regardless of the type of cue being used. This might suggest an overall ease of processing speed comprehension that comes with greater fluency.

Overall, these results were in line with both Experiments 1 and 2. More specifically, both suppletion and backfixing lines led to shorter time estimations—indicating they were perceived faster—than motion lines. Yet, suppletion and backfixing lines were not different from each other, as in Experiment 1. In addition, increase in the number of motion lines was again perceived as showing faster movement than having only two lines and this increase made them comparable to backfixing lines but not to suppletion lines, as in Experiment 2. Differently than prior experiments though, objects presented alone were comparable to motion lines when it comes to actual assessments of time duration.

The possible explanation why motion lines did not differ from cue-less objects here might come from the change in the design itself. Unlike Experiments 1 and 2, in this experiment we also had a depiction of the starting point, or the source of action, shown by extra postural cue i.e., the person throwing the ball. Since the ball was shown at the starting position first and then in the middle of air (see Figure 6), it might have seemed less “frozen” compared to objects directly presented in the middle of action without the presence of a source in other experiments. The postural cue of throwing marking the cause of action and the change in the position of the ball together might have diminished the sense of having no extra cue.

Furthermore, our results align with Hayashi et al. (2012) who showed shorter response times indexing faster speed estimates for more lines (5 or 8) compared to having a single line. We also found shorter time durations for two lines than several motion lines. In addition to the number of lines, they also examined the length of motion lines in their paradigm and observed slower estimations for shorter lines. Here we did not manipulate the length of motion lines, but the length of suppletion lines was shorter than the motion lines we used. Despite this, suppletion lines found to be the fastest, which might also strengthen the notion that they function differently than motion lines in the ways they encode distinct components of motion.

Additionally, although backfixing and suppletion lines were kept straight to capture their naturalistic context, while motion lines were curved to reflect the nature of the throwing action in this experiment, effectiveness of the former two cues over motion lines aligned with earlier results. This supports the idea that the shape of motion lines corresponds to the shape of the path, while the number and type of lines encode the speed component.

In sum, perceived fictional speed led to differences in actual assessments of time. Thus, our results do not only demonstrate motion cues vary from each other in terms of how they convey speed of movement but also the speediness of cues affects the real-life judgments of that speed.

## General Discussion

In this study we examined how motion cues convey speed of movement. In Experiments 1 and 2, we compared subjective speed ratings given for different cues i.e., motion lines trailing behind movers — either as two (Experiment 1) or also several lines (Experiment 2)—backfixing lines set in the background, and suppletion lines replacing parts of movers. We also measured response times and took into account participants’ fluency in reading comics, specifically manga. Then, in Experiment 3, we examined the duration of movement indicated by participants for movers to complete their path, again based on different cues attached to the movers. In all experiments, motion cues intensified the perceived speed compared to having no extra cue, but they differ from each other in their effectiveness in doing so. Especially suppletion lines (in all experiments) and backfixing lines (in Experiment 1 and 3) were found to be cues conveying the fastest speeds. When we compiled the ratings data, we observed that the difference in speed ratings for motion lines and backfixing lines was influenced by participants’ fluency in reading manga. We further discuss general findings below.

To begin with, motion lines were deemed to convey more speed than bare objects (Experiments 1 and 2). This finding is in accordance with Carello et al. (1986) and (Gross et al., 1991) who showed the presence of lines made the movers look like they move faster. Here we found this was also the case for objects without any additive effect of postures as our stimuli were all symmetrical objects, substantiating the effectiveness of motion lines over lack of lines. However, motion lines did not go beyond being faster than objects presented alone compared to other cues. Furthermore, they did not even differ from bare objects when the path can be clarified by other means such as the presence of source and goal (Experiment 3). Since the change in the mover’s position across panels also signals motion in comics (McCloud, 1993) and the presence of the thrower indicated the cause of object’s movement in Experiment 3’s design, the path could be inferred already without a need to have any lines. When that was the case, the advantage of having motion lines over no lines disappeared, supporting that motion lines are more an indicator of direction or path of motion but do not inherently encode fast speed per se.

We also considered the possible effect of number of motion lines on the magnitude of speed. Prior literature showed that more lines resulted in faster speed ratings (Gillan & Sapp, 2005) and shorter response times indexing faster speed estimates (Hayashi et al., 2012). Indeed, increasing the number of motion lines led to higher speed ratings compared to having only two lines. This was also reflected in actual assessments of time: participants estimated movers to complete their path in a shorter time duration if more motion lines were attached to them. Altogether, these findings suggest while mere presence of motion lines clarifies the direction, their quantity is what modulates the speed. Thus, akin to the ways that spoken languages allocate conceptual dimensions about motion into different words or morphemes (Talmy, 2000), different graphic dimensions of lines might also be tapping onto different conceptual components of motion (Hacımusaoğlu & Cohn, 2023) whether it is direction/path or speed.

While motion lines index the path being traversed, other cues do not specify directional information, such as backfixing and suppletion lines. Both of these cues depict objects in the middle of a path without clarifying the previous location of the objects and they involve “reduced iconicity”—to some extent they resemble to the blurring that appears due to fast speed in real life settings. Based on this rationale, we expected them to encode fastness inherently compared to motion lines. In line with our expectations, motion lines were judged as conveying slower speeds than suppletion lines in all experiments, pointing the function of suppletion lines to denote fast speed. Interestingly, while Hayashi et al. (2012) found that shorter length of motion lines resulted in slower speed estimates, suppletion lines having the shortest length in our design were still found to be the fastest. This again highlights the difference in the ways motion lines and suppletion lines function. Backfixing lines were also effective in denoting fast speed and surpassed motion lines in general. Thus, these findings establish that motion cues function differently and vary in their effectiveness in depicting fast speed.

Whether any increase in the number of motion lines would make them closer to backfixing and/or suppletion lines was another open question. Our findings demonstrated that the increase in the number of motion lines did not make them convey speed as fast as suppletion lines, but the difference found between backfixing lines and motion lines vanished. As mentioned, this may explain why motion lines were judged to convey speed faster or comparable to backfixing lines in Gross et al.’s (1991) findings, because in their design, they compared backfixing lines with multiple motion lines (even more than our several motion lines). Also, given that suppletion lines were still the fastest in spite of the increase in number of motion lines but backfixing lines were not, we might conclude the latter remained relatively less salient than suppletion lines in their effectiveness in depicting speed. This finding also rules out the possibility that suppletion lines simply show faster movement because they (still) have more lines than several motion lines because backfixing lines had the most lines in our experiment. The saliency of suppletion lines also aligns with our reasoning that viewers might assess the speed relatively faster when an objective viewpoint is adopted compared to backfixing lines that depict the movement from a subjective viewpoint (McCloud, 1993). Altogether, although the quantity of lines adds up on the speed component, in line with the notion “more in form = more in meaning” (Lakoff & Johnson, 1980), we can conclude it is not the only determiner of perceived speed. Several factors might operate in determining speed such as which component of motion is encoded within a cue (e.g., directionality), its reduced iconicity, and/or subjectiveness.

It is also worth noting that our findings indicated a reflection of fictive speed into actual time assessments in Experiment 3. Although we retained the duration between the starting point and the midpoint of action always constant, participants indicated the duration for the rest of the path differently based on the cues attached to the movers. Thus, fictitious motion cues influenced the time it takes for individuals to respond. Their indications were in accordance with the subjective speed ratings given in Experiments 1 and 2. Namely, participants were faster to respond to the cues judged to convey faster speeds, and slower for the ones rated as conveying slower speeds. These results suggest an embodiment of motion cues in which the static representation of speed sponsors real-life assessment of that speed.

Finally, we also assessed individual differences in fluency in reading comics and manga in all three experiments. We did not find a robust relationship between people’s fluency in reading comics and their processing of the cues in all experiments, yet in Experiment 2, exposure to manga (while growing up and currently) was found to be related to participants’ response times. They took longer time to rate the cues overall. Response times in this experiment also showed backfixing lines were processed longer compared to either type of motion lines overall, but for manga readers there was a reverse trend. This might be due to manga using backfixing lines in greater proportion than other comics, leading to manga readers having an easier time assessing these familiar cues. These findings overall are consistent with other results showing that both general and type-specific proficiencies in visual narratives modulate their processing (Cohn, 2020).

In addition, when data from Experiments 1 and 2 merged together to further explore the inconsistencies in the effect of backfixing lines, we found fluency in manga readership modulated speed ratings for some motion cues. Specifically, people who read manga rated backfixing lines as conveying faster speed while their ratings for motion lines indicated slower speed compared to people with lower fluency in manga readership. These relationships are in accordance with corpus studies showing that backfixing lines are more common in manga (Cohn, 2023) while motion lines are used across various comic styles (Hacımusaoğlu & Cohn, 2022). This prevalence might make motion lines that correspond to the paths traversed less effective as speed cues for people who are used to cues that encode faster speed. Altogether, these proficiency-related findings align with prior studies where expertise with the patterns from particular comics affect comprehension (Cohn, 2020), and might explain the mixed results for backfixing lines in the literature as discussed earlier (Gross et al., 1991).

In conclusion, motion cues differ in their effectiveness in depicting speed of motion. Though motion lines have been studied extensively, less research has been given to other cues that are suggested to be part of a broader visual lexicon (Cohn, 2013; Hacımusaoğlu & Cohn, 2023). These findings further raise questions about the origin of motion lines being purely biological or metaphorical since these accounts do not consider other cues that we investigated here. While it remains unclear how different cues vary in their effectiveness in depicting speed from a perceptual or metaphorical view, our findings are in line with premises of a visual lexicon view postulating different aspects of motion (e.g., speed vs. direction) being captured by different features of cues (Hacımusaoğlu & Cohn, 2023). As discussed, the presence of motion lines gives the direction, while their quantity in form can modulate the speed. Suppletion and backfixing lines on the other hand do not index the path traversed overtly but rather are suggested to convey speed component of motion. Also, the effectiveness of backfixing lines over motion lines in conveying speed can be modulated by people’s exposure to manga, further reinforcing the learned lexical characteristics of such motion cues. Future research can further look into the nature of the relationship found between number of lines and perceived speed (Gillan & Sapp, 2005) and use converging backfixing lines (Ito et al., 2010) as a contrast since they also have directionality i.e., clarifying the future direction. Altogether, this study provides insights into overcoming the challenges of static depiction of speed.

## Data Accessibility Statement

All data and the analyses conducted have been published at its data repository (DATAVERSE NL) and can be accessed via this link: https://doi.org/10.34894/S8LC85. The repository involves a code book file that explains what each file contains.

## Footnotes

Sample size was determined to be 30 based on prior literature (Carello et al., 1986; Gross et al., 1991) but due to the inclusion criterion of age (18+), we could only recruit 25 people for Experiment 1.
